# Improvement of the Ductility of Environmentally Friendly Poly(lactide) Composites with *Posidonia oceanica* Wastes Plasticized with an Ester of Cinnamic Acid

**DOI:** 10.3390/polym15234534

**Published:** 2023-11-25

**Authors:** Alejandro Barandiaran, Diego Lascano, Nestor Montanes, Rafael Balart, Miguel Angel Selles, Virginia Moreno

**Affiliations:** Institute of Materials Technology (ITM), Universitat Politècnica de València (UPV), Plaza Ferrándiz y Carbonell 1, 03801 Alcoy, Spain; albator@epsa.upv.es (A.B.); nesmonmu@upv.edu.es (N.M.); rbalart@mcm.upv.es (R.B.); maselles@dimm.upv.es (M.A.S.)

**Keywords:** poly(lactide), *Posidonia oceanica*, reactive extrusion, plasticizer, ductility

## Abstract

New composite materials were developed with poly(lactide) (PLA) and *Posidonia oceanica* fibers through reactive extrusion in the presence of dicumyl peroxide (DCP) and subsequent injection molding. The effect of different amounts of methyl *trans*–cinnamate (MTC) on the mechanical, thermal, thermomechanical, and wettability properties was studied. The results showed that the presence of *Posidonia oceanica* fibers generated disruptions in the PLA matrix, causing a decrease in the tensile mechanical properties and causing an impact on the strength due to the stress concentration phenomenon. Reactive extrusion with DCP improved the PO/PLA interaction, diminishing the gap between the fibers and the surrounding matrix, as corroborated by field emission scanning electron microscopy (FESEM). It was observed that 20 phr (parts by weight of the MTC, per one hundred parts by weight of the PO/PLA composite) led to a noticeable plasticizing effect, significantly increasing the elongation at break from 7.1% of neat PLA to 31.1%, which means an improvement of 338%. A considerable decrease in the glass transition temperature, from 61.1 °C of neat PLA to 41.6 °C, was also observed. Thermogravimetric analysis (TGA) showed a loss of thermal stability of the plasticized composites, mainly due to the volatility of the cinnamate ester, leading to a decrease in the onset degradation temperature above 10 phr MTC.

## 1. Introduction

In recent decades, concern about environmental pollution has increased considerably. This concern has directly impacted the polymer industry, since their use and production generate a significant negative impact on the environment [1]. This is because most of the polymers used come from fossil resources, so their production generates immense amounts of CO_2_ that contributes to increasing the carbon footprint [2]. Furthermore, most of the commercial polymers, such as poly(ethylene) (PE), poly(propylene) (PP), and poly(styrene) (PS), among others, have low or no biodegradation capacity, generating large amounts of waste for landfills [3,4]. A new trend is focused on the development of polymeric materials to replace conventional polymers, meeting requirements such as that their origin must be from renewable sources and that the material is capable of disintegrating under controlled composting conditions [5,6,7]. Among the wide range of alternatives, one can find green, or wood–plastic, composites (WPCs). WPCs are materials based on polymeric matrices combined with fillers or natural fibers [8,9]. Several polymers have been proposed in these applications, including aliphatic polyesters, such as poly(ε-caprolactone) (PCL) [10], poly(butylene succinate-*co*-adipate) (PBSA) [11], and poly(glycolide) (PGA) [12], among others. Despite being derived from fossil resources, these polymers are biodegradable, which justifies their use [13,14]. Despite the fact that the use of these polymer matrices is increasing, poly(lactide) (PLA) is the aliphatic polyester with the most industrial applications due to the need to find a balance between cost and performance. Although PLA can be obtained from fossil resources, currently it is mainly obtained from natural resources [15,16].

Poly(lactide) (PLA) belongs to the family of aliphatic polyesters. Several methods can be used to obtain PLA, the most widely used being ring-opening polymerization (ROP) of the cyclic lactide dimer. Lactide, in turn, can be obtained from the anaerobic fermentation of glucose from starch-rich products, such as potatoes, sugar cane, corn, or rice [17]. Overall, PLA has good mechanical, thermal, and biodegradation/disintegration properties [18]. But it also has significant limitations, such as low ductility and brittleness [19]. This feature is typically passed down to the compounds made using this substance. Several technical approaches have been reported in the literature to overcome this drawback and increase the ductility of these materials. Industrially, it is most common to mix PLA with other more flexible biopolymers, such as PBSA, PBS, PCL, PBAT, and PBAT, among others [20,21,22]. Also, adding natural (nanocrystal cellulose) or synthetic nanofillers to PLA is another efficient way to strengthen and toughen the material [23,24,25]. Another alternative is to add another ductile material obtained from renewable resources to increase the impact resistance or ductility of PLA, such as isoprene rubber and natural rubber, whose main component is cis-1,4-poly(isoprene), where the PLA/natural rubber blends have been shown to have higher impact strength [26]. One of the most widely used techniques is plasticization. Among the most commonly used materials in PLA plasticization, it is worth highlighting the use of oligomers of lactic acid (OLA) [27]; citric acid esters, such as acetyl tributyl citrate (ATBC) [28]; or modified vegetable oils (VOs), such as epoxidized linseed oil (ELO), maleinized linseed oil (MLO), and epoxidized soybean oil (ESBO), among others [29]. Bocqué et al. [30] suggested the potential of some biobased compounds as candidates for plasticization, including esters from cinnamic acid, coumarin-3-carboxylic acid, and 2,5-furandicarboxylic acid and their esters. Barandiaran et al. [31] have recently reported the exceptional plasticization properties of different cinnamic acid esters on PLA. They reported an increase in the elongation at break from 3.9% (neat PLA) up to values above 300% with 20 wt.% of different cinnamates, such as methyl *trans*–cinnamate, isobutyl cinnamate, allyl cinnamate, and ethyl cinnamate. Accordingly, these cinnamates also provided a noticeable decrease in the glass transition temperature (T_g_) of neat PLA located at 61.7 °C, down to values close to 36 °C; thus, showing the exceptional plasticization efficiency of these new plasticizers.

The use of lignocellulosic materials as reinforcement or fillers in thermoplastic-based composites is widespread. One of the main reasons is their abundance, which is a key factor in their high level of availability and low price. Lignocellulosic materials come from renewable resources, do not damage machinery during their processing, and have good resistance properties [32]. *Posidonia oceanica* seaweed (PO) is an aquatic plant that grows abundantly in the Mediterranean Sea [33]. *Posidonia oceanica* seaweed grows near the coast at a depth of about 40 m, and its presence is vital to prevent the effects of erosion [34]. This seaweed has three different parts: leaves, rhizomes, and scales. After its life cycle (annual), the dead leaves separate from the scales [35]. This debris tends to wash up on nearby shorelines, causing large amounts of waste to accumulate, in the form of fibrous balls, which causes a significant cleanup problem for the responsible authorities, since this waste must be removed every summer. The very fact of its abundance makes it easy to obtain, as well as being cost effective. This makes this waste plant a good candidate for the manufacture of biocomposites. Garcia-Garcia et al. [34] reported on the manufacturing of fiberboards using *Posidonia oceanica* as reinforcement in an epoxy resin matrix through thermocompression, obtaining materials with a high level of content from a natural origin (70 wt.% of *Posidonia oceanica*) and with good mechanical properties. Scaffaro et al. [36] reported on innovative composites from PLA reinforced with *Posidonia oceanica* fibers. These materials showed good elastic and flexural modulus values, due to the excellent dispersion of the fibers in the matrix due to the production process. This effect was more significant as the percentage of *Posidonia oceanica* increased.

As already stated, one of the purposes of using WPCs is to reduce the use of polymeric matrices, replacing them with natural fillers without affecting the overall properties of the composite. Unfortunately, one of the significant problems in WPC manufacturing is the low compatibility of natural fibers with polymeric matrices, due to their different polarity. This interaction plays a key role in the final properties of the material, since a good load transfer to the fiber depends on it [37]. This issue has limited their use in applications where the components are subjected to significant stresses. Several alternatives have been proposed to improve the fiber–matrix interaction, such as incorporating compatibilizing agents, or surface treatments, on the lignocellulosic fibers before the manufacturing process, among others [38,39]. Another interesting approach is reactive extrusion (REX) with low amounts of organic peroxides, such as dicumyl peroxide (DCP), benzoyl peroxide (BPO), or di-(2-tert-butyl-peroxyisopropyl)-benzene (BIB), among others [40,41,42]. DCP is one of the most widely used peroxides in the industry, as a free radical initiator in polymerization, grafting, and reactive extrusion [40]. Sari et al. [43], reported an improved interaction between pandanwangi fiber and a PE matrix using 4% DCP. An improvement in the tensile, flexural, and impact properties was obtained in this study. Ahmad and Luyt [44] observed that the reactive extrusion of PE and sisal fibers with DCP leads to the formation of grafted PE in the sisal fibers, especially in the samples with LPDE and LLDPE. Gomez-Caturla et al. [45] reported on the improved mechanical properties of bio-based PP composites reinforced with mango peel particles. Reactive extrusion with 1 phr DCP and 3 phr PP-*g*-IA (compatibilizer) led to a synergistic effect involving the matrix and fibers, causing an increase in the stiffness of the material.

Although WPCs have a promising present and future, as mentioned above, one of the main problems is their extreme brittleness and low ductility. Therefore, the present study aims to observe the effect of different amounts of plasticizer (methyl *trans*–cinnamate) on the mechanical properties of the material, emphasizing the ductile properties of PLA-based composites reinforced with *Posidonia oceanica* fibers. In addition, to improve the interaction between the fibers, the plasticizer, and the matrix, DCP is used during the extrusion process. Reactive extrusion is expected to provide improved interactions between the base polymer and the lignocellulosic filler; additionally, the presence of a carbon–carbon double bond in the cinnamate could play a key role in anchoring the plasticizer to provide improved toughness.

## 2. Experiment

### 2.1. Materials

Commercial semi-crystalline PLA of Purapol L130 grade, distributed by Corbion (Amsterdam, The Netherlands), was used. According to the manufacturer, the PLA has a density of 1.24 g/cm^3^, and an MFI (210 °C/2.16 kg) of 16 g/10 min, and a stereochemical purity > 99 (% L-isomer). *Posidonia oceanica* (PO) seaweed was collected from the Spanish Mediterranean coast in the form of fibrous balls. Dicumyl peroxide (DCP) with a molecular weight of 270.37 g mol^−1^ and a purity of more than 97.5% was used. The plasticizing agent used was a cinnamic acid ester, namely methyl *trans*–cinnamate (MTC) with a molecular weight of 162.188 g mol^−1^. The DCP and MTC were obtained from Sigma-Aldrich (Steinheim, Germany).

### 2.2. Manufacturing of PLA–Posidonia oceanica Composites

**Figure 1:**
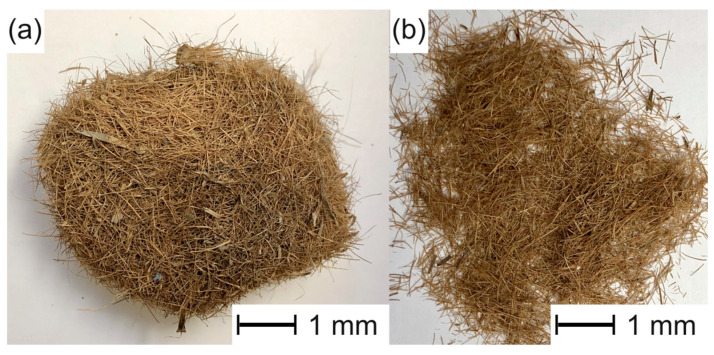
Images of (**a**) as-received fibrous ball of *Posidonia oceanica* and (**b**) *Posidonia oceanica* fibers (PO) after the cleaning process. Images with a marker scale of 10 μm.

The Posidonia oceanica (PO) fibrous balls were crushed and washed several times to remove sand and impurities (Figure 1). Then, the PO fibers obtained were dried at 65 °C for 12 h in an oven; the approximate length of the fibers was between 2 mm and 8 mm, and the aspect ratio was in the 7–10 range [34,46].

Before processing, the PLA pellets were dried at 65 °C for 12 h in an oven to avoid hydrolysis. The PLA, Posidonia fibers, and plasticizer (MTC) were manually premixed in a container for 5 min, until a homogeneous mixture was obtained (visually identifying that the plasticizer thoroughly wets the PO fibers and the PLA). Before the extrusion process, the DCP was added to the PLA–PO–MTC mixture and homogenized for 5 min to ensure a good distribution of the components.

All samples were formulated to maintain a constant percentage of 70 wt.% PLA and 30 wt.% PO fibers. The plasticizer (MTC) was incorporated in 10 and 20 phr amounts; the DCP was incorporated in 1 phr during the reactive extrusion (REX) process. The materials were coded PLA–PO–ZZMTC–DCP, where “ZZ” represents the amount (phr) of plasticizer used. The reactive extrusion and subsequent injection molding process were performed in an Xplore MC 15HT micro-compounder and an Xplore micro-injection molder IM12, respectively; both devices were supplied by Xplore Instruments BV (Sittard, The Netherlands). The temperature profile for the extrusion process was 180–190–200 °C. The PLA–PO–MTC–DCP mixture was fed into the extruder hopper manually at a slow and constant speed, ensuring that the screw speed was kept constant (100 rpm). The mixture was held in the plasticizing chamber for 2 min to ensure the homogeneity of the mix and to allow the reaction by the DCP. The injection pressure was set at 5 bar, and the injection and cooling time was 4 s. The injection barrel and mold temperature were 205 °C and 30 °C, respectively.

### 2.3. Characterization of PLA/PO Composites

#### 2.3.1. Chemical Characterization

The chemical interactions of PLA and PO, MTC, and DCP in the composites were investigated using attenuated total reflectance–Fourier transform infrared spectroscopy (FTIR–ATR). The assay was performed using a Spectrum Two FT-IR spectrometer equipped with a universal ATR accessory, both supplied by Perkin Elmer Spectrum BX (Waltham, MA, USA). Every formulation was assessed over a range of 4000–450 cm^−1^ using 36 scans, a resolution of 4 cm^−1^, and a range of 2 cm^−1^.

#### 2.3.2. Mechanical Characterization

The mechanical properties of the PLA and PLA/PO samples were analyzed under tensile, impact strength, and hardness conditions. Tensile tests were performed on a universal testing machine, model ELIB 30, supplied by Ibertest (Madrid, Spain). The tests were performed on standardized specimens (ISO 527 [47]) in a bone shape. Five samples of each formulation were tested, the results were averaged, and the standard deviation was calculated. The equipment used a 5 kN load cell, with a crosshead speed of 10 mm min^−1^. The elastic modulus E_t_, tensile strength σ_t_, and elongation at break ε_b_ were obtained from the tensile test. The impact strength test was carried out using the Charpy test. The test was performed with a Charpy pendulum, supplied by Metrotec S.A. (San Sebastian, Spain), namely a 6-J pendulum. The specimens used were unnotched, and the dimensions followed ISO 179 [48]. Five samples of each formulation were tested. The Shore D hardness was measured by a hardness tester, model 673-D, supplied by J. Bot S.A. (Barcelona, Spain). The test was performed at room temperature, following the indications in ISO 868 [49], with an applied force of about 20 N, using an indenter with an angle of 30° and a tip radius of R0.1. The measurements were performed at five different points on all the samples, with a stabilization time of 15 s.

#### 2.3.3. Morphological Characterization

Morphological analysis of the fracture surfaces obtained in the impact test on the PLA and PLA/PO parts was performed using field emission scanning electron microscopy (FESEM) using a Zeiss Ultra 55 FESEM microscope, supplied by Oxford Instruments (Abingdon, UK). The equipment used an electron voltage acceleration of 1.5 kV. The surfaces were sputter coated by applying an ultrathin layer of gold–palladium, using an EM MED20 high-vacuum coater, supplied by Leica Microsystem (Milton Keynes, UK).

#### 2.3.4. Thermal Characterization

The main thermal transitions of the PLA and PLA/PO composites were obtained using differential scanning calorimetry (DSC). The tests were performed using a DSC 821 calorimeter from Mettler Toledo Inc. (Schwerzenbach, Switzerland). The samples had weights ranging from ≈5–8 mg. High-purity aluminum crucibles with 40 µL capacity were used. The tests were performed under a nitrogen inert atmosphere (66 mL min^−1^). The samples underwent a dynamic DSC consisting of three cycles. The first cycle was performed to erase the thermal history derived from the previous transformation process; this cycle went from 25 °C to 200 °C. The subsequent cooling cycle went from 200 °C to −25 °C. The second heating cycle went from −25 °C to 300 °C. The heating and cooling rate was 10 °C min^−1^. From the second cycle, transitions such as the glass transition temperature (T_g_), cold crystallization temperature (T_cc_), melting temperature (T_m_), cold crystallization enthalpy (ΔH_cc_), and melting enthalpy (ΔH_m_) were extracted. The degree of crystallinity was calculated using Equation (1).

(1) $$\chi_{c}=\frac{∆H_{m}-∆H_{cc}}{∆H_{m}^{0}\cdot \left(1-w\right)}\cdot 100\%$$

where w is the weight fraction of the PO, and $∆H_{m}^{0}$ corresponds to the theoretical melting enthalpy of the fully crystalline PLA (93 J g^−1^) [50].

The thermal stability at high temperatures and the degradation of the PLA and PLA/PO composites were studied using thermogravimetric analysis (TGA). The tests were performed using a Mettler Toledo TGA/SDTA 851 thermobalance (Schwerzenbach, Switzerland). The samples weighed were ≈15 mg and they were placed in 70 µL sealed alumina crucibles. The samples were subjected to a heating cycle from 30 °C to 800 °C at a heating rate of 10 °C min^−1^; the test was performed under a nitrogen atmosphere, with a flow rate of 66 mL min^−1^. This test extracted parameters, such as the onset degradation temperature, T_5%_, which stands for the temperature measured when the sample loses 5% mass, and the maximum degradation rate temperature (T_max_) from the first derivative curve (DTG).

#### 2.3.5. Wettability

The surface wettability of the PLA and PLA/PO composites was studied using the water contact angle (WCA). The test was performed at room temperature with distilled water, with a droplet size of ≈1.5 µL. Droplets were deposited at different locations on the samples. This procedure was repeated eight times for each formulation. The measurements were performed with an EasyDrop-FM140 optical goniometer, supplied by KRÜSS GmbH (Hamburg, Germany).

#### 2.3.6. Dynamic Mechanical Thermal Analysis (DMTA)

The thermomechanical properties of the PLA and PLA/PO samples were evaluated using dynamic mechanical thermal analysis (DMTA). The tests were performed using a dynamic analyzer, model DMA1, supplied by Mettler Toledo (Schwerzenbach, Switzerland). The equipment works in a single cantilever for samples with certain dimensions (1 × 7 × 20 mm^3^). The samples were subjected to a heating program ranging from −50 °C to 140 °C, with a heating rate of 2 °C min^−1^. The equipment was calibrated to have a frequency of 1 Hz and a maximum shear deflection of 0.1%. This analysis allowed the obtaining of the evolution of the storage modulus (E′) and the dynamic damping factor (tan δ) concerning the temperature. 

## 3. Results and Discussion

### 3.1. Chemical Characterization

**Figure 2:**
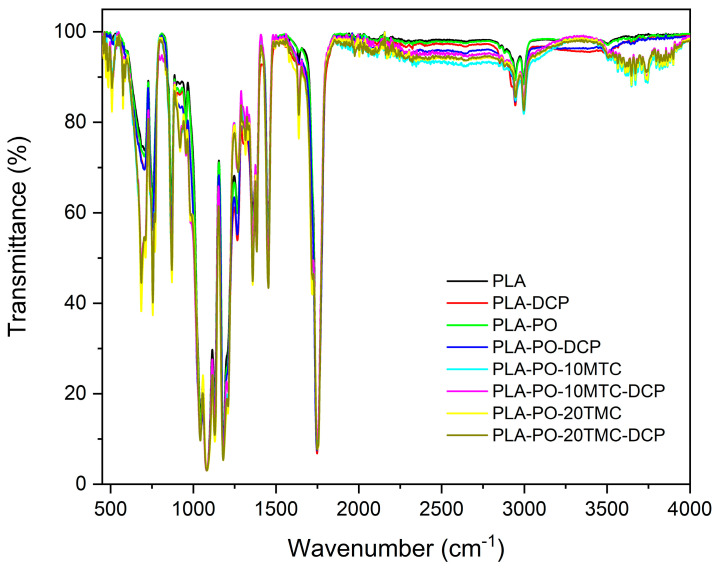
Fourier transform infrared spectroscopy (FTIR) spectra of neat PLA and PLA/PO composites with methyl *trans*–cinnamate, processed by conventional or reactive extrusion (REX).

Figure 2 shows the infrared spectrum obtained for each of the studied samples, where the functional groups’ characteristic peaks are observed. The peak appearing at 3000 cm^−1^ corresponds to the O–H and C–H bonds. The peak at 1750 cm^−1^ corresponds to the C=O bond in the carbonyl group. The peak corresponding to 1450 cm^−1^ is assigned to the C–H bond in the methyl (CH3) functional groups [51]. The region between approximately 1200 and 600 cm^−1^ is attributed to the C–C and C–O bonds. This region is known as the fingerprint region because, although the bands appearing here are the product of various types of bond vibrations, which give rise to a robust interaction between neighboring bonds, each sample has a particular spectrum. These peaks are present in all the samples, since PLA is the main component of all the mixtures [52].

Figure 2 shows that incorporating DCP, cinnamate esters, and Posidonia oceanic modifies the spectra. New peaks around 500 and 600 cm^−1^ correspond to the new bonds formed with PLA. Two noticeable peaks, around 700 and 750 cm^−1^, correspond to the C–H bonds. The peak above 900 cm^−1^ is also attributed to this type of bond. On the other hand, the peak around 1650 cm^−1^ for PO alone does not appear. Still, it does appear in the presence of the other components, being very noticeable when the corresponding ester is added [53]. Considering this peak is attributed to the ester group, it could be related to a chemical interaction between the PLA, PO, and DCP, reducing the availability of ester groups in the obtained mixtures due to the excellent miscibility between all the components. Finally, several peaks appear in the 3500 and 4000 cm^−1^ region, attributed to hydrogen bonds between the PLA and esters, since these do not appear for the PLA alone, PLA–DCP, PLA–PO, PLA–PO, PLA–PO, and PLA–PO–DCP. This effect indicates interactions between the MCT and the polymer matrix. All this justifies that the plasticizing agent, DCP, and the Posidonia interact with PLA efficiently due to the changes observed in the FTIR.

### 3.2. Mechanical Properties of PLA/PO Composites

The main mechanical properties are summarized in Table 1. Neat PLA exhibits a high elastic modulus (E_t_) of 3242.3 MPa, a high tensile strength (σ_t_) of 64.2 MPa, and a very low elongation at break with values of approximately 7.1%. These values are in agreement with the typical behavior of a brittle polymer. The reactive extrusion of PLA in the presence of DCP does not produce a perceptible change in the tensile properties (σ_t_ and ε_b_) of the PLA–DCP samples, maintaining values similar to those of neat PLA; however, a slight increase in E_t_ is observed, reaching values of 3576.4 MPa. As is well known, DCP is commonly used to produce long-chain branching (LCB). The use of this peroxide induces the crosslinking of PLA chains through the biomolecular recombination of radicals to form C–C bonds, as suggested by Takamura et al. [54]. As a result, a slight increase in the stiffness of the material is obtained. The addition of PO fibers causes an increase in the stiffness of the composites, resulting in an increase of 30% in the E_t_ values (4231.5 MPa). When compared to neat PLA, it is also possible to appreciate the rise in the brittleness of the material, observing a decrease of 17% and 37% in the σ_t_, and ε_b_ values, respectively. This effect is related to the low interaction of the natural fibers with the PLA, primarily due to the different nature of the PO fibers and the PLA matrix. PO fibers typically behave as a lignocellulosic material, with a high hydrophilic character. PLA, in turn, has a high hydrophobic behavior, resulting in the low compatibility of these composites and a low ability to transmit stresses [55]. These results are similar to those presented by Haddar et al. [56], where incorporating Posidonia fibers into a HDPE matrix resulted in materials with high stiffness and tensile strength, but this harmed the elongation at break and impact strength. These results are comparable to those presented by Vijayasekaran et al. [57], which were concerned with the manufacturing of PE-based composite materials reinforced with Posidonia fibers. This study observed the low compatibility of Posidonia fibers with the PE matrix. They reported that the incorporation of the fibers impaired the elongation at break and impact strength due to the poor load transmission.

Reactive extrusion with DCP in the PLA–PO–DCP formulation has no significant effect on the tensile strength (53.9 MPa) and elongation at break (4.1%), as they are practically similar to those of the material without DCP (PLA–PO). A similar effect was observed by Rytlewski et al. [58], in PLA composites reinforced with flax and hemp fibers. They attempted to improve the fiber/matrix interaction using reactive extrusion with DCP and they concluded that the effect was negligible for small amounts of DCP (<0.5 wt.%). Despite this, a decrease in the elastic modulus was observed, reaching values of 3119.1 MPa, similar to neat PLA.

As mentioned above, different amounts of methyl *trans*–cinnamate were incorporated into PLA–PO composites to increase the ductile behavior of the developed composites. It is observed that by adding 10 phr of methyl *trans*–cinnamate in the PLA–PO–10MTC and PLA–PO–10MTC–DCP composites, the E_t_ is 3290 MPa and 3619.8 MPa, respectively, and the values of σ_t,_ and ε_b_ are approximately 39 MPa and 4.6%, respectively, for the two composites. It is found that this amount of methyl *trans*–cinnamate (10 phr) does not have a significant influence on the mechanical properties. However, a decrease of approximately 26% in the tensile strength was observed compared to the composites processed using conventional extrusion instead of reactive extrusion (REX) (PLA–MTC, PLA–PO–10MTC). This decrease may be due to the different deformation capacities of the fibers and the polymer, together with the matrix continuity disruption produced by the fibers, which promotes microcrack formation and growth under stress conditions [59]. It should be noted that, theoretically, methyl *trans*–cinnamate is considered a suitable plasticizer for PLA as reported previously, since the solubility parameter of PLA is 22.66 MPa^1/2^, and that of methyl *trans*–cinnamate is 19.36 MPa^1/2^ [31]. This effect is negligible in our case, as no significant change in the tensile properties was observed. This may be caused by the fact that the amount of plasticizer used was not sufficient to trigger a plasticizing effect in the matrix.

The most remarkable results were obtained when incorporating 20 phr of methyl *trans*–cinnamate (PLA–PO–20MTC). This composite shows a considerable decrease in stiffness concerning materials without plasticizer (PLA–PO), where elastic modulus values of 1092.7 MPa and a tensile strength of 16.4 MPa were observed. The reduction in E_t_ can be attributed to the plasticization effect generated by methyl *trans*–cinnamate, since plasticizer molecules are located between the PLA chain, resulting in a decrease in the intermolecular forces, weakening its stiffness and, at the same time, increasing its ductility [60]. The reduction in tensile strength may be due to the aggregates of fibers that form due to the Van der Waals forces of attraction [61].

The most noticeable effect is the dramatic increase in the elongation at break with values of 21.6%, corresponding to an improvement of more than 200% compared to neat PLA. These results are fascinating and promising since one of the drawbacks of a WPC is its high brittleness, which is much more pronounced with brittle polymer matrices such as PLA. The increase in the material’s flexibility is due to the plasticization caused by the methyl *trans*–cinnamate in the PLA matrix, derived from the presence of ester groups in the plasticizer that helps form hydrogen bonds with ester groups in PLA [30,62]. The results are comparable to those presented by Dominguez-Candela et al. [63], concerning PLA composites reinforced with lignocellulosic particles (e.g., chia seeds) and plasticized with maleinized chia oil (MCO) and epoxidized chia oil (ECO). An elongation at break of 13% and 16% were observed for MCO and ECO, respectively. In addition, a compatibilization effect with the lignocellulosic filler was observed, in which the oxirane and maleic anhydride groups present in ECO and MCO, respectively, reacted with the terminal hydroxyl groups in the lignocellulosic filler and PLA chains, resulting in materials with balanced mechanical properties.

Reactive extrusion (REX) with DCP (PLA–PO–20MTC–DCP) provides an increase of 171% in the elastic modulus and a decrease of 68% in the tensile strength compared to composites with the same composition processed using conventional extrusion (PLA–PO–20MTC). These results can be attributed to the fact that the incorporation of the fibers and the DCP produces an increase in the crystallinity of the composite, where crystallites are formed at the interface between the fibers and the PLA, which favors the formation of microcracks [58]. However, an increase of 44% in the elongation at break is observed in composites processed using REX compared to conventional extrusion. This effect may be related to the plasticization effect generated by the methyl *trans*–cinnamate in the PLA.

Regarding impact strength, the Charpy test results for the PLA and PLA/PO composites are summarized in Table 1. PLA presents impact strength values of 38.9 kJ m^−2^, and the reactive extrusion (REX) with DCP causes an increase of 28% when compared to neat PLA. This effect may be related to the crosslinking effect that DCP provides [54]. The addition of Posidonia oceanica fibers causes a remarkable decrease in the impact strength, compared to neat PLA, up to values of 26.5 kJ m^−2^. As mentioned above, the low interaction of natural fibers with polymeric matrices (PLA) causes a stress concentration phenomenon, which results in a low stress transfer capacity, causing a decrease in the impact energy absorption capacity [64]. Reactive extrusion (REX) with DCP (PLA–PO–DCP) causes an increase in the impact strength values. This effect may derive from the fact that DCP, on the one hand, causes the crosslinking and/or branching phenomena on the matrix chains, as mentioned above. On the other hand, REX can lead to an increase in the interaction between the *Posidonia oceanica* fibers and the matrix, by grafting the polymer chains onto the surface of the lignocellulosic fibers. These results have the same trend as those reported by Rojas-Lema et al. [65], whose results showed an improvement in the impact strength values through reactive extrusion with DCP, resulting in improved interactions between a high-density polyethylene matrix, reinforced with lignin particles.

By incorporating the plasticizer in different concentrations in the PLA–PO–10MTC and PLA–PO–20MTC materials, impact strength values of approximately 30 kJ m^−2^ are obtained. These results suggest a slight increase in toughness when compared to the values obtained for unplasticized composites (PLA–PO) (26.5 kJ m^−2^). The plasticizing capacity of MTC in the PLA matrix may be responsible for this outcome. These results are similar to those observed by Arrieta et al. [27]. They reported an exceptional improvement in toughness by adding more than 10 wt.% oligomers of lactic acid (OLA) to a PLA matrix.

Reactive extrusion with DCP produces a decrease in the impact strength of the PLA–PO–10MTC–DCP and PLA–PO–20MTC–DCP composites, obtaining values close to 17 kJ m^−2^. As previously mentioned, reactive extrusion with DCP of PLA–PO composites leads to an increase in crystallinity. This directly affects the material’s behavior during the fracture process, promoting microcrack formation and growth [58].

The Shore D hardness of neat PLA is 82.6. Due to the standard deviation, no noticeable effect can be identified by incorporating *Posidonia oceanica*, and methyl *trans*–cinnamate, processed using conventional or reactive extrusion with DCP.

### 3.3. Morphological Characterization

**Figure 3:**
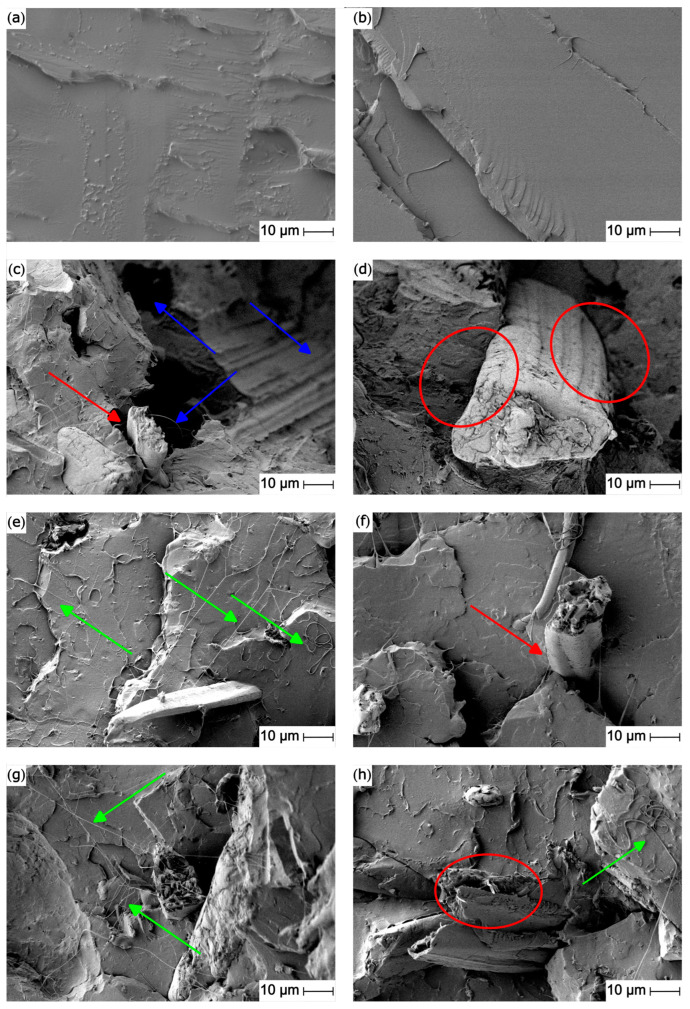
FESEM images of the fracture surface of the (**a**) PLA, (**b**) PLA–DCP, (**c**) PLA–PO, (**d**) PLA–PO–DCP, (**e**) PLA–PO–10MTC, (**f**) PLA–PO–10MTC–DCP, (**g**) PLA–PO–20MTC, (**h**) PLA–PO–20MTC–DCP. Images were taken at 1000×, with a marker scale of 10 μm.

Figure 3 shows the FESEM images of the fracture surfaces obtained in the Charpy test of the PLA and PLA/PO composites. Figure 3a shows the fracture of the neat PLA, which shows a flat, smooth, and soft surface, without any evidence of plastic deformation. This fracture is typical of a brittle polymer [66]. Figure 3b shows how REX with DCP modified the surface fracture, changing from a brittle fracture (typical of PLA) to a surface with higher roughness, which is clear evidence of slight plastic deformation during fracture. These images corroborate the results obtained in the mechanical impact tests, where an increase in the energy absorption capacity was observed, due to the increase in the crystallinity of the compounds. This type of surface is similar to those presented by Tábi et al. [67], concerning PLA composites plasticized with an oligomer of lactic acid (OLA).

In Figure 3c, it can be observed how the incorporation of *Posidonia oceanica* fibers causes significant disruptions in the PLA matrix continuity. The presence of small gaps between the fibers and the surrounding matrix (red arrows) indicates poor fiber/matrix interactions. This low fiber/matrix interaction is due to the different nature of the fibers (highly hydrophilic) and the matrix (highly hydrophobic) [68]. The phenomenon of premature detachment or the pulling out of the fibers from the matrix is also evident (blue arrows). This effect can be attributed to the disordered distribution of the fibers in the matrix. These two phenomena limit the appropriate transmission of stresses from the matrix to the reinforcing fibers, causing a stress concentration effect, and leading to a drop in the mechanical ductile properties. These results are similar to those presented by Jawaid et al. [37], who observed that the random distribution of nonwoven fibers caused a loss of mechanical properties in the fabrication of polymeric composites reinforced with fibers, due to the stress concentration effect generated by the fibers.

Reactive extrusion (REX) with DCP causes a clear improvement in the fiber/matrix interaction (Figure 3d). This is appreciable in the decreasing gap between the fiber and the surrounding PLA matrix (red circle). This improvement in the interaction translates into an increase in the overall performance of the materials, as can be seen in the resistive properties.

The effect of methyl *trans*–cinnamate incorporation is also appreciable (Figure 3e–h). Plasticizing filaments can be observed, in addition to heterogeneous surfaces, demonstrating plastic deformation. The appearance of these filaments suggests an increase in the material’s ductility, which is more appreciable in Figure 3e,g (green arrows) corresponding to the samples with 10 phr and 20 phr of methyl *trans*–cinnamate. REX with DCP shows a decrease in the filaments, suggesting a reduction in plastic deformation and flexibility (Figure 3f,h), which explains the drop in the impact strength values. However, an improvement in the fiber/matrix interaction (red circle) is appreciated, leading to a combined effect of sample failure. On the one hand, physical deformation of the PLA entangled chains is present. On the other hand, the rupture of the crystals formed at the periphery of the Posidonia oceanica fibers decreases the toughness properties, as suggested by Pawłowska et al. [69].

### 3.4. Thermal Characterization

**Figure 4:**
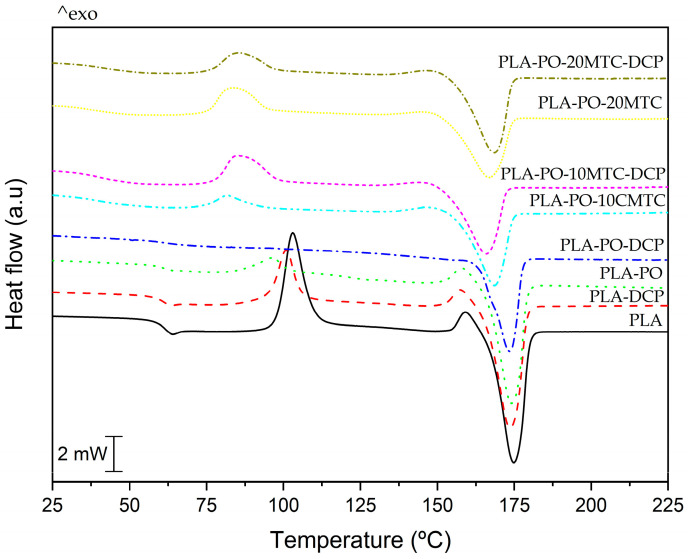
DSC curves of the second heating run involving neat PLA and PLA/PO composites with methyl *trans*–cinnamate processed using conventional or reactive extrusion (REX).

Figure 4 shows the calorimetric curves (DSC) corresponding to the second heating cycle of the neat PLA and PLA/PO composite samples. Table 2 summarizes the main thermal transitions obtained from the DSC test. PLA exhibits thermal transitions typical of a semi-crystalline material, such as a glass transition temperature, T_g_ of 61.6 °C, a cold crystallization process with a maximum peak temperature, T_cc_ located at 103.1 °C, and a melting peak temperature, T_m_ of 174.2 °C. Concerning the glass transition temperature, it is observed that the incorporation of *Posidonia oceanica* fibers and REX with DCP in PLA–DCP, PLA–PO, and PLA–PO–DSC materials does not cause any significant effect on the T_g_, maintaining an approximate value of 60 °C, which is very similar to that of neat PLA. As mentioned above, one of the drawbacks of PLA-based composites is their low ductility, and low impact properties. To address this disadvantage, plasticizers are often incorporated. The effectiveness of these plasticizers depends on several factors, the main ones being miscibility, molecular weight, amount of plasticizer, and others [70]. As is known, the plasticizer is placed preferentially in the amorphous regions of semi-crystalline polymers, so the effectiveness of a plasticizer is determined by its ability to decrease the glass transition temperature [71]. The effect of methyl *trans*–cinnamate on the thermal properties of composites is impressive. Concerning the glass transition temperature, a significant decrease was observed. In particular, it was observed that after incorporating 10 phr and 20 phr, the T_g_ ranges between 37 °C and 41 °C, thus indicating a good plasticization effect, and corroborating what was previously observed in the FESEM images. These results are similar to those presented by Xu et al. [60], concerning plasticized PLA with acetyl tributyl citrate (ATBC). They observed a decrease in the T_g_ of neat PLA, down to 40 °C in PLA composites reinforced with nano fibrillated celluloses (NFCs), plasticized with 10 wt.% ATBC. Similarly, Sharma et al. [72], obtained PLA materials reinforced with halloysite nanotubes (HNT) and plasticized with triethyl citrate (TEC), with glass transition temperatures close to 47 °C. This effect is because the plasticizer molecules are inserted between the PLA chains, causing an increase in the free volume, which contributes to enhancing the chain mobility [73].

Concerning the cold crystallization temperature, the unplasticized samples show a slight decrease in the peak temperature of this process, T_cc_ values, changing from 103 °C for the neat PLA to approximately 96 °C (PLA–PO). This behavior is similar to that presented by Johari et al. [74]. Despite incorporating fillers, they tend to accelerate the cold crystallization process in the matrix, such that the low fiber loading does not cause a significant perturbation in the movement of the matrix chains, so the overall effect is negligible. Similarly, a slight decrease in T_cc_ is observed in composites processed using REX with DCP, as suggested by Simmons and Kontopoulou [75]. This decrease is because PLA undergoes degradation during the hydrolysis process.

The impact of the plasticizer is also observed in the shift by the T_cc_ to lower temperatures. It is observed that methyl *trans*–cinnamate causes a decrease in the T_cc_ of all the composites, down to values between 81–85 °C. This decrease is correlated with the reduction in T_g_. The effect of the decline in T_cc_ is that, on the one hand, the plasticizer facilitates the movement of PLA molecules, allowing rearranging and crystallization to occur more efficiently and with less energy [76]. On the other hand, the presence of *Posidonia oceanica* can act as a nucleating agent promoting the early formation of crystallites. The same phenomenon was identified by Aguero et al. [77], by adding diatomaceous earth to a PLA matrix. They reported that the presence of diatomaceous earth decreased the T_cc_ from 119 °C (pure PLA) to 115 °C, but a more pronounced decrease was achieved by adding maleinized linseed oil (MLO), which produced a decrease in T_cc_ down to lower values of 108 °C.

The degree of crystallinity (χ_c_) was calculated considering the second heating cycle. The values are summarized in Table 2. PLA shows a degree of crystallinity of 22.9%. REX with DCP and the addition of *Posidonia oceanica* causes a considerable increase in PLA crystallinity. The PLA–DCP and PLA–PO materials present an χ_c_ of 42.6% and 64.6%, respectively. This increase is due to several overlapping phenomena, such as the rise in M_w_, caused by the REX with DCP, as suggested by Torres-Giner et al. [78], and to the nucleating effect caused by the presence of *Posidonia oceanica* lignocellulosic fibers [79]. Concerning the plasticized composites, an apparent increase is observed in the χ_c_, where values between 28–45% were reached. This agrees with the suggestions by Garcia-Garcia et al. [76], where the extra mobility that the chains receive after plasticization helps the PLA chains to rearrange into a packed structure, thus leading to an increase in crystallinity. However, the disordered distribution of *Posidonia oceanica* fibers causes the formation of these structures to be disordered [65,79]. Helping the increase in crystallinity values. These results are similar to those presented by Arrieta et al. [80], who reported that better particle dispersion of cellulose nanocrystals caused an increase in the degree of crystallinity in a PLA–PHB blend.

**Figure 5:**
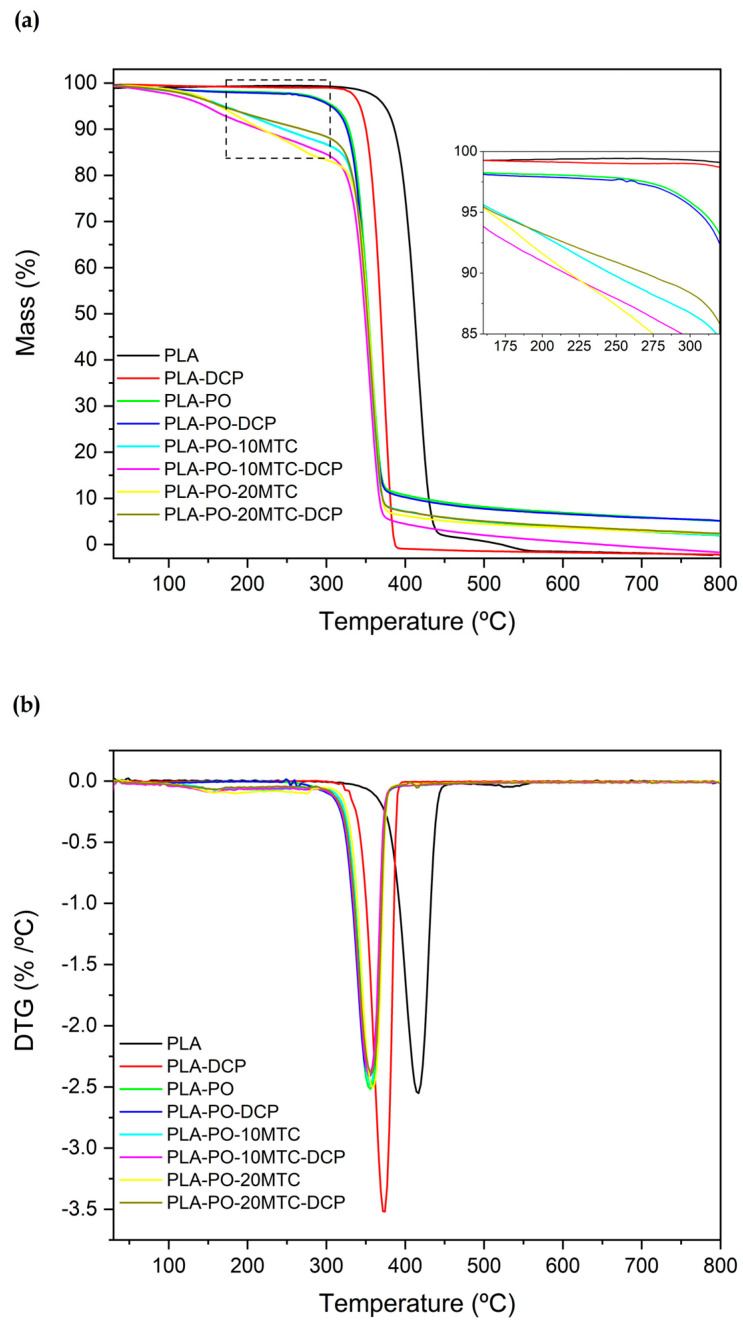
Comparative plots of (**a**) TGA curves and (**b**) first derivative DGT curves of neat PLA and PLA/PO composites with methyl *trans*–cinnamate, processed using conventional or reactive extrusion (REX).

The study of the high-temperature thermal stability of the PLA and PLA/PO composites was carried out using thermogravimetric analysis (TGA). Figure 5 shows the thermograms obtained from the TGA. In addition, Table 3 summarizes the main parameters obtained from the study, which are the representative temperature at the onset of degradation (T_5%_), the maximum degradation rate temperature (T_max_), and the percentage of the residual mass (%_mass_) at 800 °C. As shown in Figure 5a,b, PLA has a T_5%_ and a T_max_ of 374.3 °C and 417 °C, respectively. The thermal degradation process occurs in a primary step and a residual step. The former, between 350–440 °C, is associated with the cleavage of the PLA main chain due to the breakup of the ester groups. This stage is characterized by a mass loss of about 97% [66]. The second stage occurs between 440 °C and 550 °C, where the degradation of the residual chains in the previous process takes place.

It is evident that reactive extrusion with DCP negatively affects the thermal stability of PLA, with both the T_5%_ and T_max_ decreasing to 342.3 °C and 374.3 °C. This decrease can be attributed to the fact that in the crosslinking process that PLA undergoes due to REX with DCP, tertiary carbons are generated and introduced into the PLA structure, which are more prone to thermal degradation [81]. These results are similar to those found by Yamoum et al. [82], where the incorporation of ethoxylated bisphenol A dimethacrylates (Bis-EMAs) as a crosslinking coagent in a chemically crosslinked PLA (in the presence of DCP), caused a slight loss in the thermal stability of the composites. The PLA–PO and PLA–PO–DCP composites show an accelerated onset of degradation, with a drop of about 67 °C at T_5%_ (307.7 °C), compared to neat PLA. *Posidonia oceanica* presents a similar structure to that of any lignocellulosic compound [34]. Thermogravimetric degradation of lignocellulosic materials is characterized by a first mass loss at about 100 °C, corresponding to the evaporation of residual moisture, followed by the decomposition of hemicellulose, cellulose, and pectin in the range of 220 to 450 °C, and finally the degradation of lignin at about 550 °C [83]. The loss of thermal stability at low temperatures by the materials (PLA–PO and PLA–PO–DCP) can be attributed, on the one hand, to the fact that organic *Posidonia oceanica* components such as hemicellulose exhibit low thermal stability and tend to degrade at low temperatures, as suggested by Ortiz-Barajas et al. [84]. These results are analogous to those presented by Seggiani et al. [46], where in PO-reinforced PHA composites, the incorporation of PO caused an acceleration in the onset of degradation. On the other hand, at around 100 °C, DCP decomposes, accelerating the degradation process of the materials due to presence of free radicals and less thermally stable carbons formed during REX [58].

Regarding the plasticized composites, an evident loss in thermal stability is observed. In fact, the TGA characteristic curves change to a several step degradation process. The first stage occurs at 100 °C, with a mass loss of 2–2.5% corresponding to the residual moisture in *Posidonia oceanica*. The next step occurs in the range between 170 °C and 200 °C and may be ascribed to plasticizer volatilization, due to its low molecular weight, as happens with many other monomeric plasticizers [85]. Despite this, the plasticizer’s efficiency was not compromised. This effect was most notable for the pieces with 20 phr, which showed clear plasticization, as evidenced by the good mechanical properties and DSC analysis. These results are analogous to those presented by Ivorra-Martinez et al. [86], concerning plasticized PLA materials with DBI, who reported a plasticizer loss of up to 11 wt.% during processing, due to the high volatility of the DBI. Despite this, excellent plasticization was observed, as reflected in the mechanical properties. Also, Gomez-Caturla et al. [87], managed to plasticize PLA effectively using diethyl l-tartrate (DET). However, they observed that due to the low molecular weight of DET, it volatilized at temperatures below 200 °C, causing a decrease in the onset temperature of degradation in the materials. This effect was more accentuated as the amount of DET increased. Finally, the last stage of degradation is related to the decomposition of the PLA chains and is found between 320–380 °C.

A decrease in the maximum degradation temperature of the composites concerning PLA is observed. This reduction is more notorious for the composites loaded with *Posidonia oceanica*, this temperature being around 350 °C; this may be caused by the premature decomposition of the PO fibers and the slight volatilization of methyl *trans*–cinnamate. Finally, a residual mass of approximately 5% was observed in the PO-loaded materials due to the formation of ash.

### 3.5. Wettability Characterization

**Figure 6:**
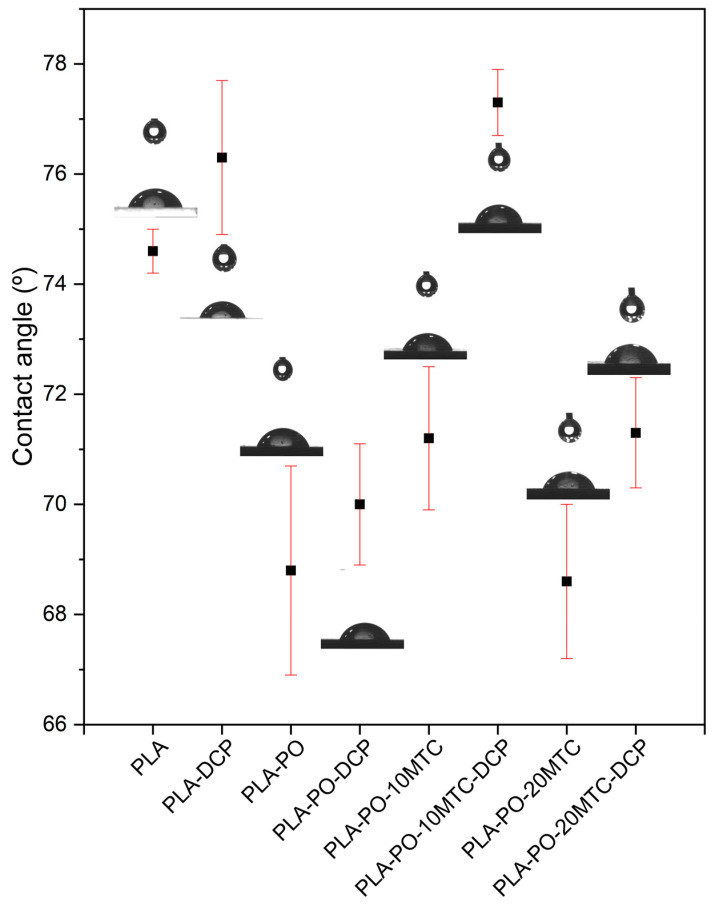
Water contact angle (WCA) of neat PLA and PLA/PO composites with methyl *trans*–cinnamate, processed using conventional or reactive extrusion (REX).

Figure 6 shows the variation in the water contact angle (WCA) measured on the surface of the PLA and PLA/PO composites. Generally materials are usually classified as hydrophobic or hydrophilic. Vogler [88] proposed a threshold to categorize this affinity: a material with a WCA > 65° presents a hydrophobic character and, consequently, a material with a WCA < 65° presents a hydrophilic nature.

Neat PLA presents a water contact angle of 74.6°, presenting a high hydrophobic character, which agrees with the literature [89]. As expected, the REX with DCP slightly increases the WCA, showing values of 76.3° for the PLA–DCP samples. This effect can be attributed to the crosslinking generated by the DCP in the PLA chains [54], causing the hydrophobic behavior to increase due to decreased space between the chains, limiting the interaction with water. Incorporating *Posidonia oceanica* fibers into the PLA matrix causes a 7.8% decrease in the WCA concerning neat PLA, generating an increase in the hydrophilic character of the materials, which was expected due to the intrinsic hydrophilicity of lignocellulosic materials. This effect is due to two factors. On the one hand, *Posidonia oceanica* fibers present a large number of hydroxyl groups that generate a good affinity with water through hydrogen bonding [79,90]. On the other hand, due to the low fiber/matrix interaction, voids are generated between these elements, facilitating water insertion into the matrix. The higher fiber/matrix interaction generated by reactive extrusion with DCP causes a slight increase in the hydrophobic character of the composites, as can be observed in the WCA values (70°) of the PLA–PO–DCP sample.

In the plasticized composites (PLA–PO–10MTC and PLA–PO–20MTC), a WCA of 71.2° and 68.6°, respectively, was observed, where the presence of methyl *trans*–cinnamate does not cause significant changes in the hydrophobic behavior of the materials when compared to unplasticized composites.

Finally, as previously mentioned, REX with DCP helps to improve both the fiber/matrix interaction and the crosslinking/branching of PLA. This effect is reflected in the high WCA values of the composites, a consequence of which, the hydrophobic character of the samples (PLA–PO–10MTC–DCP and PLA–PO–20MTC–DCP) increases.

### 3.6. Dynamic Mechanical Thermal Characterization

**Figure 7:**
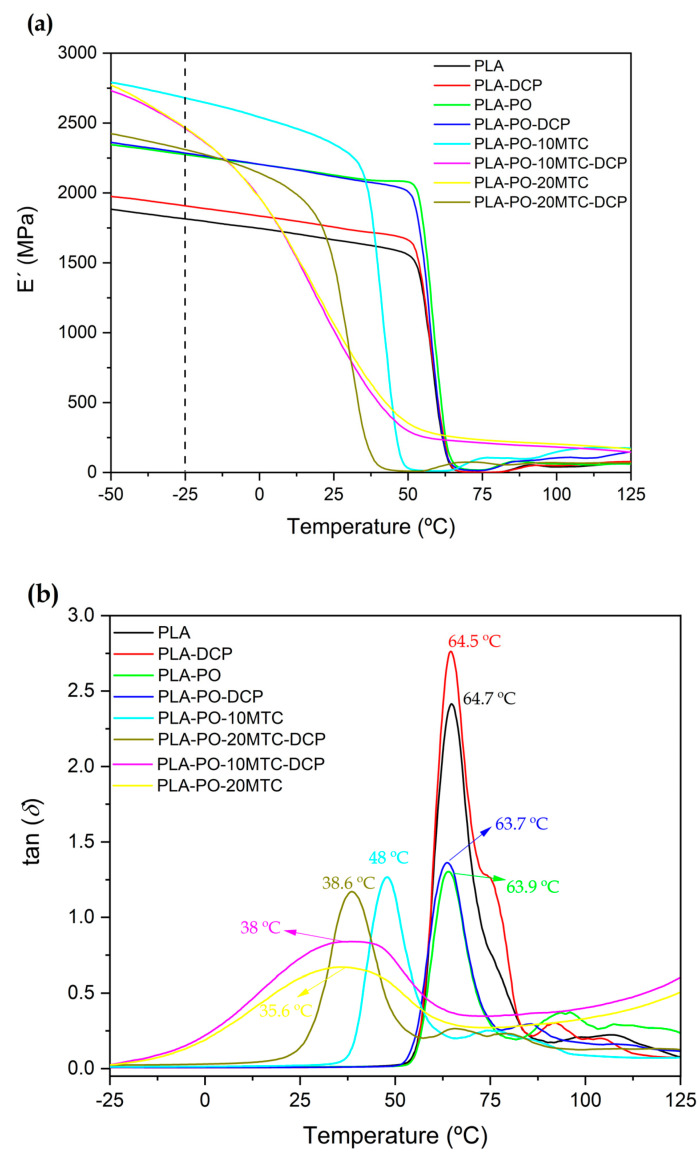
Plot evolution of (**a**) the storage modulus (E′) and (**b**) the dynamic damping factor (tan δ) of neat PLA and PLA/PO composites with methyl *trans*–cinnamate, processed using conventional or reactive extrusion (REX).

Figure 7 shows the dynamic mechanical thermal analysis (DMTA) curves of the neat PLA and PLA/PO composites. Figure 6a compares the evolution of the storage modulus (*E*′) as a function of the temperature. The DMTA behavior of neat PLA serves as a base for the comparison. At low temperatures, PLA shows a high stiffness marked by high *E*′ values. In this work, PLA offers values of *E*′ ≈ 1814 MPa (−25 °C). As the temperature increases from 50 °C to 75 °C, *E*′ shows a drop of approximately two orders of magnitude. This pronounced drop in the storage modulus is related to a relaxation (*α*), attributable to the glass transition temperature [91].

After analyzing the PLA/PO composites at low temperatures, it was observed that the apparent storage modulus in the composites with *Posidonia oceanica* fibers increase, far exceeding the 1814 MPa of neat PLA. This increase in the storage modulus can be attributed to the *Posidonia oceanica* fibers having an effect on the PLA matrix, resulting in materials with higher stiffness. These results are similar to those presented in the literature for biomaterials reinforced with lignocellulosic particles [91,92]. It should be noted that reactive extrusion with DCP does not lead to a significant change in the storage modulus.

Furthermore, incorporating different amounts of methyl *trans*–cinnamate causes a shift in the *E*′ curves towards lower temperatures. As mentioned above, the plasticizing effect produced by this cinnamate helps the mobility of the polymeric chains, which in turn, decreases the stiffness of the materials since the ductile behavior is improved [93]. It can be observed that the addition of 10 phr of methyl *trans*–cinnamate does not lead to a significant plasticizing effect in the composites and corroborates the results observed in regard to the mechanical properties, in which evident plasticization was observed in composites containing 20 phr of cinnamate. However, increasing the amount of cinnamate (20 phr) leads to a higher plasticizing effect. The noticeable shift in the curves towards lower temperatures corroborates this. These results agree with those reported by Erdem and Doğan [94], concerning PLA composites with hydromagnesite, additionally plasticized with acetyl tributyl citrate (ATBC). A decrease in the storage modulus values was obtained, being more intense with increasing amounts of plasticizer. This effect is due to an increase in the segmental movement of the chains caused by plasticization.

The estimation of the glass transition temperature of the compounds was carried out under the criterion of the maximum peak in the dynamic damping factor, tan *δ* vs. T (Figure 7b). Figure 7b shows that neat PLA presents a T_g_ of ≈ 64.7 °C, corroborating the results obtained in the calorimetric study. The presence of *Posidonia oceanica* fibers in the unplasticized composites does not lead to any change in the T_g_, remaining practically constant (63.7–64.5 °C). However, the incorporation of methyl *trans*–cinnamate causes a noticeable decrease in the T_g_ values of the plasticized materials, reaching T_g_ values below 40 °C. As mentioned above, the effectiveness of a plasticizer is determined by its capacity to decrease the glass transition temperature [95]. It was observed that the decrease in the T_g_ of the material obtained by the material with 10 phr cinnamate (PLA–PO–10MTC) was less pronounced, which is in agreement with the mechanical and thermal results obtained. Nevertheless, T_g_ values below 50 °C are obtained, comparable to those obtained with other plasticizers [76,96]. The results obtained in this study are relevant since, generally, reinforced biopolymers usually have low flexibility, and the herein developed composites with *Posidonia oceanica* offer interesting ductile properties. These results agree with those presented by Barandiaran et al. [31], who reported a decrease in the glass transition temperature of PLA (67 °C) to 37.8 °C by adding 20 wt.% of methyl *trans*–cinnamate. Similarly, Ivorra-Martinez et al. [86] demonstrated that incorporating 20 wt.% dibutyl itaconate (DBI) into a PLA matrix resulted in glass transition temperatures below 40 °C. Moreover, this was reflected in the increased ductility of the materials.

## 4. Conclusions

The investigation covers the manufacturing of new biocomposites with PLA matrix and *Posidonia oceanica* (PO) fibers. The composites were fabricated using conventional and reactive extrusion (REX) in the presence of dicumyl peroxide (DCP) and subsequently injected. The effect of different amounts of methyl *trans*–cinnamate as a plasticizer on the behavior of the materials was studied. The presence of *Posidonia oceanica* fibers caused an increase in the stiffness of the materials, as well as an increase in the brittleness, a decrease in the ductility, and an increase in the impact strength. The loss of properties was due to the low interaction of *Posidonia oceanica* with PLA, as observed in the FESEM images, which led to a stress concentration effect. Reactive extrusion with DCP improved the fiber/matrix interaction and produced a crosslinking/branching effect in the PLA chains, causing an increase in the impact strength. Moreover, REX with DCP also provided some grafting of the polymer chains onto the *Posidonia oceanica* surface, which was reflected by the smaller gaps between the fibers and the surrounding matrix. When incorporating 20 phr of cinnamate as an environmentally friendly plasticizer, an excellent plasticizing effect was observed. The methyl *trans*–cinnamate caused a considerable increase in the elongation at break until exceeding values of 30%, and an increase in the ductile behavior of the materials was also observed, as evidenced by the formation of plasticizing filaments observed in the FESEM study, due to plastic deformation. Simultaneously, the glass transition temperature of neat PLA at 65 °C was remarkably reduced to 40 °C, thus showing the high plasticization efficiency of methyl *trans*–cinnamate. The reactive extrusion (REX) of PLA/*Posidonia oceanica* and methyl *trans*–cinnamate composites in the presence of DCP caused an increase in the crystallinity of the materials and a loss of thermal stability at high temperatures. This was evidenced by the decrease in the onset degradation temperature (T_5%_) obtained using thermal analysis. The reduction in T_5%_ was more pronounced in the plasticized materials due to the partial loss of the plasticizer during processing, as pointed out by the TGA results. Dynamic mechanical thermal analysis (DMTA) also revealed the improvement on the ductile properties provided by methyl *trans*–cinnamate in combination with the reactive extrusion with dicumyl peroxide.

## Figures and Tables

**Table 1:** Summary of the mechanical properties of the neat PLA and PLA/PO composites plasticized with methyl *trans*–cinnamate processed using conventional or reactive extrusion (REX).

Code	Tensile			Shore D Hardness	Impact Strength (kJ m^−2^)
	Elastic Modulus, E_t_ (MPa)	Strength, σ_t_ (MPa)	Elongation at Break, ε_b_ (%)		
PLA	3242.3 ± 89.9	64.2 ± 3.7	7.1 ± 0.3	82.6 ± 1.0	38.9 ± 6.1
PLA–DCP	3576.4 ± 71.3	62.8 ± 2.9	7.8 ± 0.9	82.1 ± 1.5	49.8 ± 4.6
PLA–PO	4231.5 ± 27.0	53.5 ± 1.4	4.5 ± 0.4	83.9 ± 1.6	26.5 ± 4.7
PLA–PO–DCP	3118.1 ± 90.4	53.9 ± 1.5	4.1 ± 0.2	84.4 ± 1.0	34.3 ± 2.6
PLA–PO–10MTC	3292.0 ± 78.2	39.6 ± 0.4	4.4 ± 0.2	82.5 ± 0.8	31.3 ± 2.6
PLA–PO–10MTC–DCP	3619.8 ± 80.1	39.9 ± 0.8	4.8 ± 0.5	81.5 ± 1.6	18.1 ± 1.6
PLA–PO–20MTC	1092.7 ± 29.6	16.4 ± 1.2	21.6 ± 2.3	79.0 ± 2.8	30.9 ± 8.7
PLA–PO–20MTC–DCP	2961.4 ± 78.5	5.1 ± 0.5	31.1 ± 12.1	80.3 ± 2.9	17.0± 2.1

**Table 2:** Main thermal transitions and properties of neat PLA and PLA/PO composites with methyl *trans*–cinnamate processed using conventional or reactive extrusion (REX), obtained using differential scanning calorimetry (DSC).

Code	T_g_ (°C)	T_cc_ (°C)	ΔH_cc_ (J g^−1^)	T_m_ (°C)	ΔH_m_ (J g^−1^)	χ_c_ (%)
PLA	61.6 ± 1.2	103.1 ± 1.2	27.9 ± 0.5	174.2 ± 4.5	49.3 ± 1.0	22.9 ± 0.3
PLA–DCP	60.9 ± 1.0	100.8 ± 1.8	14.8 ± 0.3	173.0 ± 2.4	54.4 ± 0.9	42.6 ± 0.9
PLA–PO	61.4 ± 1.1	96.3 ± 1.4	5,3 ± 0.1	173.5 ± 3.1	47.3 ± 0.7	64.6 ± 1.3
PLA–PO–DCP	61.4 ± 1.1	-	-	173.1 ± 3.5	31.9 ± 0.5	48.9 ± 0.8
PLA–PO–10MTC	39.6 ± 0.8	81.6 ± 1.6	5.2 ± 0.1	168.2 ± 3.2	34.8 ± 0.7	45.4 ± 0.8
PLA–PO–10MTC–DCP	39.3 ± 0.6	85.0 ± 1.4	14.5 ± 0.2	165.5 ± 3.1	34.9 ± 0.6	31.4 ± 0.6
PLA–PO–20MTC	37.3 ± 0.7	83.2 ± 1.4	12.9 ± 0.2	174.3 ± 3.0	31.4 ± 0.6	28.4 ± 0.5
PLA–PO–20MTC–DCP	41.6 ± 0.7	85.3 ± 1.5	10.4 ± 0.2	168.2 ± 3.2	36.6 ± 0.9	40.2 ± 0.8

**Table 3:** Main thermal degradation parameters of neat PLA and PLA/PO composites with methyl *trans*–cinnamate processed using conventional or reactive extrusion (REX), obtained using differential scanning calorimetry (DSC).

Code	T_5%_ (°C)	T_deg_ (°C)	%_mass_
PLA	374.3 ± 7.5	417 ± 6.3	0.0 ± 0.1
PLA–DCP	342.3 ± 5.1	374.3 ± 5.6	0.0 ± 0.1
PLA–PO	307.7 ± 4.9	355.7 ± 5.7	5.12 ± 0.1
PLA–PO–DCP	307.7 ± 4.3	353 ± 5.1	5.13 ± 0.1
PLA–PO–10MTC	169.0 ± 2.3	355.7 ± 4.9	1.96 ± 0.1
PLA–PO–10MTC–DCP	147.7 ± 2.8	355.7 ± 7.1	0.0 ± 0.1
PLA–PO–20MTC	163.7 ± 2.6	358.3 ± 6.8	2.26 ± 0.1
PLA–PO–20MTC–DCP	166.3 ± 2.5	358.3 ± 5.7	2.36 ± 0.1

## Data Availability

Data are contained within the article.
